# Effect of Seed Hydropriming on the Elongation of Plumule and Radicle During the Germination Process and Changes in Enzyme Activity Under Water-Deficient Conditions

**DOI:** 10.3390/plants13243537

**Published:** 2024-12-18

**Authors:** Ju-Young Choi, Young-Hwan Ju, Ayaka Nakamichi, Seong-Woo Cho, Sun-Hee Woo, Jun-Ichi Sakagami

**Affiliations:** 1 The United Graduate School of Agricultural Sciences, Kagoshima University, Kagoshima 890-0065, Japan; jychoi8519@gmail.com (J.-Y.C.); joh7598@naver.com (Y.-H.J.); 2 Graduate School of Agriculture, Forestry and Fisheries, Kagoshima University, Kagoshima 890-0065, Japan; k2414988@kadai.jp; 3 Department of Smart Agro-Industry, College of Agriculture and Life Sciences, Gyeongsang National University, Jinju 52725, Republic of Korea; chsw78@gnu.ac.kr; 4 Department of Crop Science, Chungbuk National University, Cheongju 28644, Republic of Korea; shwoo@chungbuk.ac.kr; 5 Faculty of Agriculture, Kagoshima University, Kagoshima 890-0065, Japan

**Keywords:** hydropriming, *Oryza sativa*, germination, germination phase

## Abstract

Hydropriming rice seeds effectively improve the germination percentage, shortens the germination period, and promotes seedling growth. The impact of seed hydropriming is to speed up growth under dry soil conditions, thereby avoiding drought damage. This study analyzes the effect of hydropriming on morpho-physiological changes in the water uptake of rice seeds using “Kasalath” and “Nipponbare” under water-deficit conditions. Upon exposure to osmotic stress, both varieties showed delays in the time to reach germination. In addition, all germination phases exhibited reductions in the activity of alpha-amylase and total soluble sugar by osmotic stress; however, in all germination phases of the hydroprimed seeds, the activity and contents of those were significantly increased, resulting in increased size of the coleoptile, plumule, and radicle. In hydroprimed seeds, “Kasalath” was superior to “Nipponbare” in the ratio of the water-deficit-to-well-watered conditions for all traits related to germination, which may have been attributable to hydropriming having a greater effect on “Kasalath”. Interestingly, Primed “Kasalath” had a lower level of α-amylase, despite the having a higher content of total soluble sugars than primed “Nipponbare”.

## 1. Introduction

Climate change is causing prolonged droughts on several continents around the world and is expected to increase by 36.64–45.40% in 2050 [1]. Drought stress poses a significant threat to global food security, especially in countries where rice (*Oryza sativa* L.) is the main cultivated crop. Since more than half of the world’s population relies on rice as an integral part of its diet, it is important to understand and mitigate the effects of drought stress on rice production [2]. Drought stress in critical growth stages, such as germination and the early seedling stage, can severely impair yield and quality [3].

Seed germination can be divided into three phases reflecting the absorption of water: rapid water absorption (phase 1), plateau of water absorption (phase 2), and beginning of radicle (phase 3) [4]. Absorption of water by the seeds helps the embryos to develop before germination while increasing amylase activity and fructose and glucose contents and decreasing sucrose content. When rice seeds develop, large compound starch grains in the endosperm disintegrate into small starch grains, in which small holes form and many cavities between the embryo and the endosperm appear. The rapid degradation of starch by α-amylase can induce rapid germination [5,6]. The process of starch degradation, which is regulated by α-amylase, β-amylase, and maltase, is a basic metabolic process that supplies energy and respiration during germination. Among these, although α-amylase is the most important enzyme, and its activity is highly related to the rates of rice germination and seedling growth [7,8,9], it is particularly susceptible to drought stress, and a decrease in its activity reduces the content of total soluble sugar and the respiration rate of rice seeds and seedlings, which in turn lowers the germination rates under drought stress [10]. It is thus essential to develop effective strategies to improve the ability to degrade starch and the germination performance under drought stress.

It has been reported that drought stress severely affects the germination performance of rice [11]. Seed priming is an effective way of alleviating the damaging effects of such stress. Priming can potentially enhance the resistance of plants when exposed to stress, and signal transmission is rapidly activated upon subsequent exposure to stress [12]. Seed priming, involving the pretreatment of seeds to control the process of seed hydration in the radicle pre-germination phase 1 to trigger multiple metabolic processes, contributes to improving cell membrane integrity [11] by facilitating respiratory metabolism [13] and enhancing activation of reactive active oxygen removal systems [14], especially under adverse conditions such as drought stress. In addition, it improves the process of starch degradation by increasing α-amylase activity and total soluble sugar content. In particular, it has been proven that hydropriming rice seeds is an effective, practical, and easy technique to improve the germination rate, shorten the germination period, and promote seedling growth [15] and crop yield [16]. Priming of seeds has also been reported to hasten the development of radicles and plumes [17].

Because polyethylene glycol (PEG) can cause osmotic stress while reducing the water availability of plants, it has been widely used to evaluate the response to drought in many experiments [18,19,20,21,22]. Although a PEG-induced water-deficit condition is different from drought in soil, the use of PEG is considered to be extremely effective and reproducible in clarifying the reaction due to insufficient water supply in plants [20].

We examined two different varieties, “Kasalath” and “Nipponbare”, because the drought tolerance of “Kasalath” and the drought sensitivity of “Nipponbare” have been widely evaluated in previous papers [19,23,24]. In addition, out of the 59 rice World Core Collections, “Kasalath” and “Nipponbare” were selected as the most resistant and susceptible varieties to dry soils based on intercultural comparative experiments. The purpose of this study is to compare different phenotypes of rice varieties in terms of physiological changes during the germination process, especially changes in the organs involved in producing enzymes and in the growth of the coleoptile, plumule, and radicle, in order to clarify the effects of hydropriming on rice seeds from biological and morphological perspectives. In addition, the effect of hydropriming on the response under osmotic stress was also studied.

## 2. Results

### 2.1. Germination

**Figure 1:**
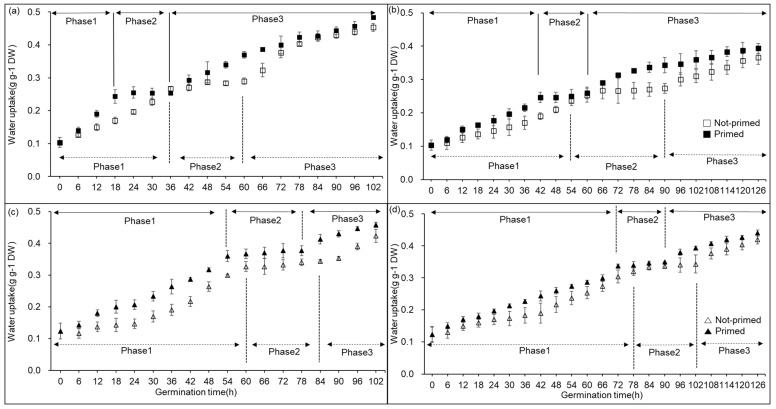
The changes in the water uptake in rice seeds of two varieties during seed germination. (**a**,**b**) represent “Kasalath”, and (**c**,**d**) represent “Nipponbare”. (**a**,**c**) represent well-watered conditions, and (**b**,**d**) represent osmotic stress conditions during seed germination. The bars in (**a**–**d**) represent standard error (S.E.). Each germination phase of 1, 2, and 3 represents imbibition, plateau, and post-germination, respectively.

**Figure 2:**
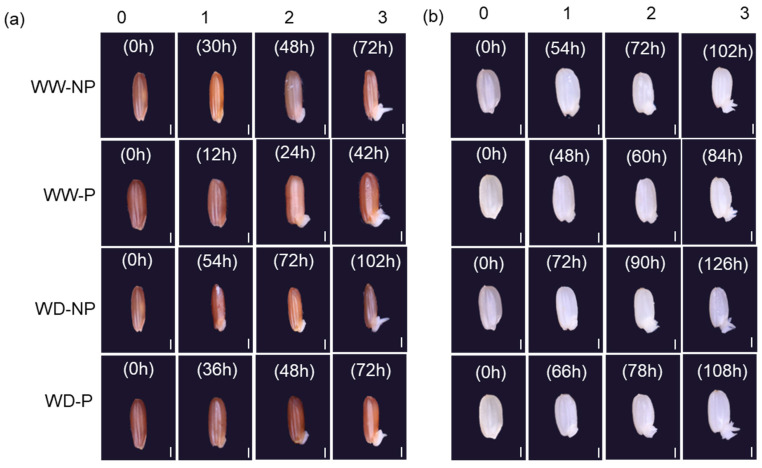
Morphological and structural changes in each phase for non-primed (NP) and primed (P) in “Kasalath” (**a**) and “Nipponbare” (**b**) under well-watered (WW) and water-deficit treatments (WD, 15% PEG6000). The 0 represents before water imbibition and 1 represents stages with the rapid absorption of water, 2 represents the stage of a plateau in water content, and 3 is the stage of radicle appearance. Scale bar = 1 mm.

To determine the germination stages of the two varieties, “Kasalath” and “Nipponbare”, we analyzed the dynamic changes in the water uptake of germinating seeds. We identified three associated phases: rapid absorption of water by seeds (phase 1), a plateau of water content (phase 2), and the initiation of germination when radicles pass through the seed coat (phase 3) (Figure 1 and Figure 2). Priming of the two varieties significantly reduced the time required for them to reach germination phases 1, 2, and 3 under well-watered and water-deficit conditions. Meanwhile, the time for each germination phase was increased by osmotic stress irrespective of whether the seeds were primed. However, primed seeds germinated more rapidly than non-primed ones. In addition, both varieties exposed to osmotic stress had decreased final germination percentages. However, the final germination percentage of primed seeds was statistically significant in both well-watered and osmotic stress conditions.

The results of T50 and MGT showed a significant interaction of V × E, which decreased in intensity in both varieties under water-deficit conditions (Table 1). In addition, T50 and MGT showed a significant P × E interaction, which was reduced in intensity for primed seeds compared with that for non-primed seeds under water-deficit conditions. In “Kasalath”, T50, MGT, and GU decreased by 24.84%, 22.19%, and 35.59% under water-deficit conditions, while the corresponding values in “Nipponbare” were 20.35%, 17.02%, and 15.05%, respectively. Primed seeds of both varieties under well-watered and water-deficit conditions showed significant reductions in T50, MGT, and GU. In “Kasalath”, T50, MGT, and GU decreased by 22.30%, 15.22%, and 21.92% under well-watered conditions but by 12.00%, 10.25%, and 14.89% in “Nipponbare”, respectively. The non-priming-to-priming ratio showed a higher effect under water-deficit conditions than under well-watered ones in both varieties.

### 2.2. Morpho-Physiological Changes Without and with Hydropriming of Seeds of Two Rice Varieties

**Figure 3:**
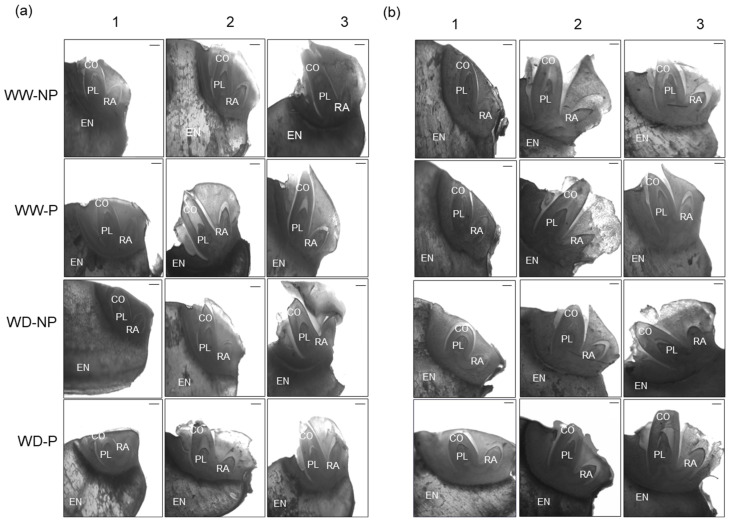
Longitudinal sections of “Kasalath” (**a**) and “Nipponbare” (**b**) embryos. Changes in well-watered (WW) and water-deficit treatment (WD, 15% PEG6000) conditions with not-primed (NP) and primed (P) according to each phase. The 1 represents the phase with the rapid absorption of water, 2 is the stage of a plateau in water content, and 3 represents the stage of radicle appearance. EN: endosperm; CO; coleoptile; PL: plumule; RA: radicle. Scale bar = 100 μm.

Figure 3 presents the structural changes of embryo and endosperm in phases 1, 2, and 3 with and without hydropriming of both varieties in the well-watered and water-deficit conditions.

**Figure 4:**
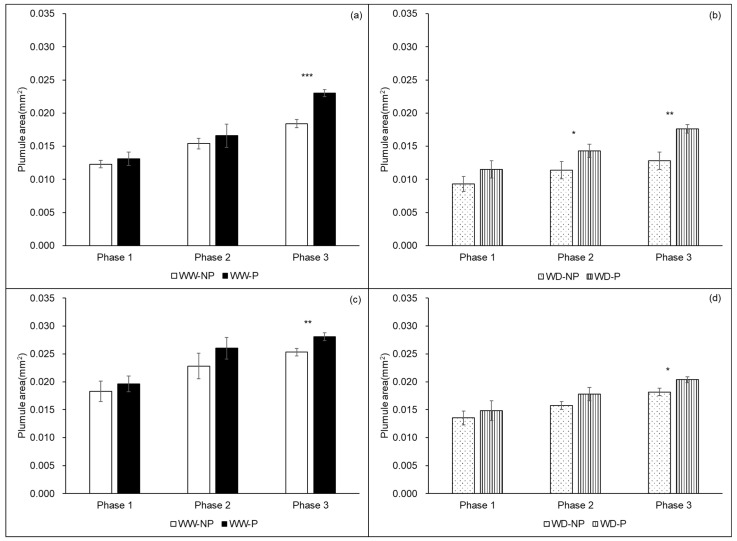
Comparison of the area of a plumule in the two varieties (see Figure 2). (**a**,**b**) represent “Kasalath” and (**c**,**d**) represent “Nipponbare”. The bars in (**a**–**d**) represent standard error (S.E.). The differences in the effect of seed priming on the plumule area of seeds germinated under control (well-watered, WW) and water-deficit (WD, 15% PEG6000) conditions were evaluated (for each step of seed germination separately) using Welch’s *t*-test. *, **, and *** indicate a significant difference (*p* < 0.05, *p* < 0.01, and *p* < 0.001, respectively).

In germination phases 1, 2, and 3, exposure to water-deficit stress in both varieties decreased the coleoptile and radicle. However, these variables were increased in the two primed varieties under both well-watered and water-deficit stress conditions compared with the corresponding levels without priming (Figure 3 and Table 2). In phase 1 and phase 2, there was a significant V × E interaction regarding the size of the coleoptile of both varieties under water-deficit stress (Table 2). In phase 3 of the coleoptile, priming treatment was noticeably increased in both varieties under water-deficit stress affected by the P × E interaction. The V × P × E interaction had a significant effect at phase 2 of the radicle of both rice varieties in the water-deficit and well-watered conditions; the first phase of the radicle was increased for both varieties primed. At phase 3 of the radicle, it was significantly affected by the interaction of V × E and P × E and was still lower than in the control. The radicle of “Kasalath” increased significantly compared with that of “Nipponbare” in phases 1, 2, and 3. The effects of priming on the “Kasalath” coleoptile under well-watered conditions were 18.35%, 13.28%, and 14.20%, respectively, in phases 1, 2, and 3. The corresponding effects in “Kasalath” exposed to water-deficit conditions were 22.33%, 25.29%, and 27.42%, respectively. Meanwhile, such effects of priming in “Nipponbare” in phases 1, 2, and 3 of germination under well-watered conditions were 10.21%, 4.28%, and 9.02%, respectively, while under water-deficit conditions, the values were 14.15%, 10.24%, and 20.30%, respectively. The effects of priming on the “Kasalath” radicle under well-watered conditions were 34.94%, 52.00%, and 45.67% in phases 1, 2, and 3, respectively, while the corresponding values under water-deficit stress conditions were 32.12%, 39.57%, and 44.43%, respectively (Figure 4). In addition, the effects of priming on the “Nipponbare” radicle under well-watered conditions were 10.22%, 16.64%, and 19.03%, while under water-deficit stress, the corresponding values were 28.30%, 27.00%, and 19.26% at phases 1, 2, and 3, respectively. In the two varieties under well-watered and water-deficit stress conditions, priming had a greater effect on radicles than on coleoptiles.

The plumule decreased in all germination phases 1, 2, and 3 of both varieties exposed to water-deficit stress, but priming increased this variable (Figure 4b,d). In the plumule of “Kasalath”, priming treatment had effects of 6.27%, 8.00%, and 24.93% in the well-watered conditions and 22.73%, 24.93%, and 34.47% in the water-deficit conditions, respectively. Meanwhile, the corresponding effects in “Nipponbare” were 6.96%, 14.06%, and 11.16% in phases 1, 2, and 3 of germination under well-watered conditions, and 9.63%, 13.14%, and 12.07% under water-deficit stress, respectively. Under well-watered conditions (Figure 4a), the priming treatment at phase 3 was significantly higher at phase 2 and phase 3 of the “Kasalath” plumule in water-deficit stress (Figure 4b). However, for the plumule of “Nipponbare”, the significance was high only at phase 3 under well-watered and water-deficit conditions (Figure 4c,d). The increases in the coleoptile, radicle, and plumule were greater in “Kasalath” than in “Nipponbare”.

### 2.3. Changes in Total Starch Content, α-Amylase Activity, and Total Soluble Sugar Content

The total starch contents in phase 1 of germination of the two rice varieties were significantly reduced upon exposure to water-deficit stress (Table 3). Those in phases 2 and 3 also decreased upon exposure to water-deficit stress, in which the P × E and V × P × E interactions were significant. Hydropriming reduced the starch contents in well-watered and water-deficit conditions. Overall, α-amylase activity decreased in both rice varieties upon exposure to water-deficit stress, which was markedly affected by the V × E interaction. However, there was no significant difference in α-amylase activity between NP and P in phase 1 in “Nipponbare” under well-watered conditions. However, α-amylase activity of “Kasalath” in phase 1 was significantly increased in P treatment compared with that in NP treatment under well-watered conditions. α-Amylase activity also showed a significant P × E interaction in phases 2 and 3. Hydropriming markedly increased α-amylase activity under water-deficit and well-watered conditions. Moreover, total soluble sugar content showed a significant V × E interaction in all phases (Table 4). Osmotic stress significantly reduced total soluble sugar content at all phases in both varieties. Interestingly, in phases 1, 2, and 3, the total soluble sugar content of “Kasalath” was markedly increased by hydropriming in the well-watered conditions. For the total soluble sugar content of the two rice varieties, pronounced V × P and V × E interactions were observed at phase 2 and phase 3. Each variety exhibited the effects of hydropriming. The hydropriming effect was identified in the well-watered and water-deficit conditions, with both varieties showing a significant effect. In addition, total soluble sugar was significantly increased by priming compared with that without priming under well-watered water-deficit conditions in both varieties.

**Figure 5:**
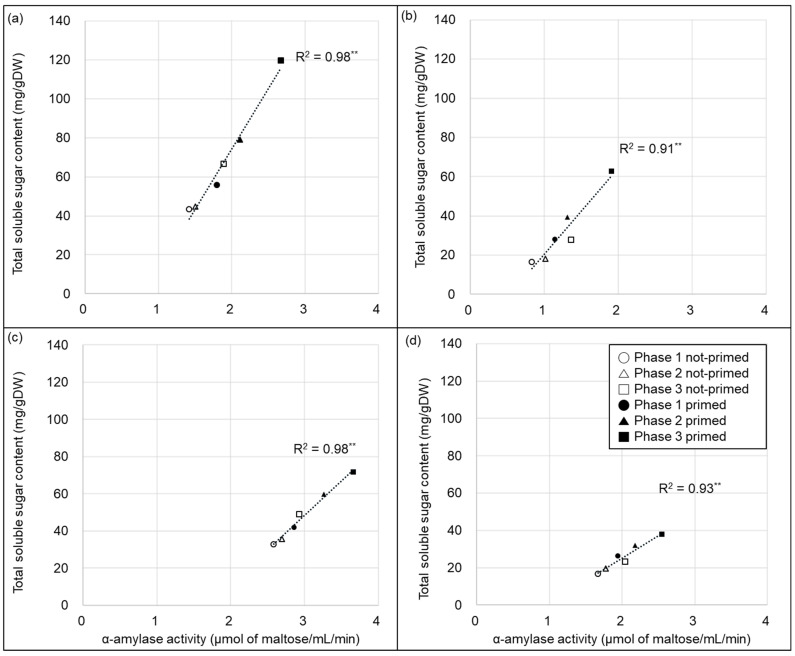
Relationship between α-amylase activity and total soluble sugar content of the two varieties. (**a**,**b**) represent “Kasalath” and (**c**,**d**) represent “Nipponbare”. (**a**,**c**) represent well-watered conditions and (**b**,**d**) represent water-deficit conditions (15% PEG6000). ** indicates significant difference at the 0.01 level (*p* < 0.01).

α-Amylase activity and total soluble sugar were highly significantly positively correlated in both varieties, in both well-watered and water-deficit conditions (Figure 5a–d).

## 3. Discussion

This study tested the hypothesis that hydropriming could induce resistance to osmotic stress during germination using 15% PEG 6000. Some studies have evaluated at 30% PEG concentration [25], but the concentration was set at 15% because no germination of susceptible varieties was observed when 30% PEG concentration was tried.

The germination of rice seeds is divided into three phases. Initially, dry seeds rapidly absorb water until the seed tissue is fully hydrated (phase 1). Then, limited water absorption occurs during phase 2, and in phase 3, water absorption increases in association with the completion of germination. The most important phase in germination is phase 2. This phase involves various cellular and biochemical events, such as DNA repair and translation of stored and newly synthesized mRNAs. In phase 2, metabolic and cellular activities increase. During germination, clarification of whether embryonic cells reenter the cell cycle or remain stationary is important in determining seedling formation [26].

Compared to non-primed seeds, hydroprimed seeds improved traits related to germination, such as T50, MGT, and GU, regardless of the well-watered conditions in the rhizosphere (Figure 1, Table 1). In addition, the effect of hydropriming was higher in conditions where water supply was limited than in conditions where sufficient water supply could be provided in the rhizosphere (Table 1). Nakao et al. [27] reported that hydropriming of seeds was effective in promoting growth in soil-drying conditions and reducing germination time, which was consistent with our findings. When seeds were subjected to the stress of water scarcity, the plumule, radicle, and coleoptile experienced reduced growth compared to adequate water conditions. However, it was found that primed seeds improved organ growth during the germination process and germinated faster than non-primed seeds (Table 2, Figure 3 and Figure 4). In conditions of moisture restriction in the rhizosphere, the effect of seed hydropriming on germination was more pronounced in the drought-tolerant variety “Kasalath’ than in the drought-sensitive variety “Nipponbare”. As described above, it was considered that the drought-tolerant varieties were more drought-resistant by the seed priming treatment than the drought-sensitive varieties. In addition to the progression of metabolic processes by seed priming, this enhancement of drought tolerance is closely related to the acceleration of the elongation of the plumule and radicle and the increase in their growth.

Starch is a major storage compound that is synthesized in rice endosperm. It serves as the main source of carbohydrates during growth at the germination and seedling stages. α-Amylase is an enzyme that breaks down starch to produce sugar. As germination proceeds, the seed increases the production of α-amylase to ensure that there is sufficient energy to promote growth. This allows starch to be converted to sugar, providing energy [28]. In this study, the total starch content in phase 1 decreased in both varieties upon exposure to water-deficit stress (Table 3). There was a significant V × P × E interaction for the total starch content in phase 2 and phase 3, and this variable was affected by hydropriming in both varieties in the well-watered and water-deficit conditions. Farooq et al. [29] reported that α-amylase activity is highly correlated with high seed germination rate, better seedling growth in the early phases of growth, and stress tolerance. In our study, the α-amylase activity following phases 1, 2, and 3 was significantly reduced under the water-deficit conditions but increased in primed “Kasalath” and “Nipponbare”. In addition, this study demonstrated the significant interactions of V × P, V × E, and P × E for all phases 1, 2, and 3 for total soluble sugars (Table 4). The increase in total soluble sugars induced by hydropriming at all phases was more pronounced in “Kasalath” than in “Nipponbare” exposed to water-deficit stress (Table 4). From Table 3 and Table 4 in combination, the increases in α-amylase and total soluble sugar by priming were greater in “Kasalath” than in “Nipponbare” under well-watered and water-deficit conditions. Priming is associated with a series of interconnected biochemical changes, such as enzyme activation and synthesis of growth promoters. Rehydration induces changes at the cellular level, such as in cell division, nucleic acid and protein synthesis, ATP production, and activation of DNA repair mechanisms. Protein, carbohydrate, and lipid mobilization enzymes are also activated [30,31]. In the primed seeds of “Kasalath”, the starch degradation process was enhanced under water-deficit stress conditions by the lower α-amylase activity and large amount of total soluble sugar. This is also related to improved coleoptile, plumule, and radicle performance of primed rice seeds under water-deficit stress [32]. It has also been reported that priming helps to maintain uniform growth of α-amylase activity and soluble sugar content [33]. Interestingly, the content of α-amylase in germination phases 1, 2, and 3 under well-watered or water-deficit conditions was higher in primed or non-primed “Nipponbare”, but the total sugar content was much higher in phases 1, 2, and 3 in “Kasalath”. This observed result may indicate the large effect of priming on “Kasalath” as well as the high energy metabolism. In addition, the shorter time to reach each germination phase due to priming in “Kasalath” might have been caused by this energy metabolism.

## 4. Materials and Methods

### 4.1. Seed Sources

We used two rice varieties in this study, “Kasalath” and “Nipponbare”, obtained from the Japan National Agriculture and Food Research Organization. We have already tested 59 varieties of the World Core Collection for their tolerance to drought and priming effect, “Kasalath” and “Nipponbare” were selected as the most effective and weakest priming effect varieties, respectively (unpublished). The seeds of all varieties used in this study were grown and harvested in 2021 in the paddy fields of Kagoshima University and then stored at a low temperature of 4 °C to maintain germination activity. Because this experiment was conducted in 2024, physiologically, it is expected that the dormancy has been removed during this period, and a high germination percentage was obtained in the actual preliminary test, so it can be asserted that there is no problem with seed dormancy in this study. Both varieties had an initial germination percentage exceeding 95% at 28 °C within 72 h after imbibition. High-quality seeds (specific density > 1.13) were used in this study. The initial seed moisture content was <13.3% (on a dry weight basis), as measured using a Riceter grain moisture meter (Kett Electric Laboratory, Tokyo, Japan).

### 4.2. Seed Priming Treatment

Hydropriming involved immersing seeds in distilled water at 28 °C and air-drying them at this same temperature until they reached their initial dry weight. Pre-germination tests were carried out using filter paper immersed in distilled water, and the appropriate immersion time was checked by incubation at 28 °C. Optimal conditions were set as priming treatment starting 6 h before the first germination was observed, with an immersion time of 18 h being selected.

### 4.3. Experimental Design

The experiment was conducted for 5 days in an SIB-35 incubator (Sanyo Corporation, Tokyo, Japan) at a temperature of 28 °C. The following treatments were performed via a completely randomized design: no priming/well-watered, no priming/water-deficit, hydropriming/well-watered, and hydropriming/water-deficit. The osmotic stress was applied by administering PEG 6000. Some studies have evaluated 30% PEG to better evaluate germination tolerance to low water potentials [25], but in the pre-experiment, the drought-susceptible variety, “Nipponbare”, did not germinate until 120 h after imbibition when 30% PEG was applied. Additionally, previous studies showed that rice seed germination and seedling growth are severely inhibited upon germination in 15% PEG [34]. Therefore, osmotic stress here involved a PEG 6000 concentration of 15% (−0.5 MPa), while the well-watered condition involved the provision of distilled water. The 15% PEG 6000 solution and the distilled water were changed daily.

Seeds of similar size were kept in water at a depth of 1 to 2 cm in a plastic box with a cover (280 mm length, 160 mm width, and 400 mm depth). Observations were performed every 6 h, up to 126 h, which were divided into phases 1, 2, and 3 of germination.

Three replicates (50 seeds × 3 biological replicates) were used to determine the three steps. Another three replicates (50 seeds × 3 biological replicates) were used to measure the properties of germination growth. A further three replicates were used to measure α-amylase activity (300 mg of seeds × 3 biological replicates), as well as total soluble sugar content (50 mg of seeds × 3 biological replicates) and total starch content (100 mg of seed × 3 biological replicates). The final three replicates (5 seeds × 3 biological replicates) were used to perform microscopic analysis of each of the three phases. The replicates were selected at random for all measurements in the present study.

#### 4.3.1. Germination Assay and Germination Test

Germination was measured every six hours after imbibition until the final germination was observed using the following formula:

Germination Percentage (GP) = (Germinated seeds/Total No. of seeds) × 100(1)

According to the time of final germination, the water uptake content of the seeds was analyzed to determine the phase of seed germination every six hours after imbibition using the following formula:

Water uptake (g/g) = (W_2_ − W_1_)/W_1_ (2)

where W_2_ represents the total seed weight (including the weight of dry seeds and imbibition water) after imbibition, and W_1_ represents the dry seed weight before imbibition [4].

The germination time to 50% germination (T50), mean germination time (MGT), germination uniformity (GU), and germination percentage (GP) were calculated according to the following formula:

(3) $$T50=t_{i}+(N/2-n_{i})*(t_{j}-t_{i})/(n_{j}-n_{i})$$

where *N* is the final number of seeds that germinated, while *n_i_* and *n_j_* are cumulative numbers of seeds that germinated as determined by counts at consecutive time points *t_i_* and *t_j_,* respectively, when *n_i_ < N*/2 *< n_j_* [27].

(4) MGT=∑(Gt∗Tt)/∑Gt

where *Gt* is the number of germinated seeds on Day *t*, and *T_t_* is the time corresponding to *Gt* in days.

(5) $$GU=\sqrt{\sum_{i=1}^{k}n_{i}(t_{i}-\bar{t}{)}^{2}/(\sum_{i=1}^{k}n_{i}-1)}$$

where $\;\bar{t}$ is the mean germination time; *t_i_* is the time between the start of the experiment and the *i*th observation (day or hour); *n_i_* is the number of seeds germinated in time *i*; and *k* is the last time of germination [27].

#### 4.3.2. Anatomical Analysis

Seeds sampled at each phase (1, 2, and 3) were fixed to pieces, sectioned at a thickness of 0.7 mm on a microtome (plant microtome MTH-1, Nippon Medical & Chemical Instruments Co., Ltd., Osaka, Japan), and immediately placed on glass slides. They were then stained in 3% Toluidine blue for 5 min. They were subsequently immersed in distilled water for 5 min and then drained. Each sample was photographed under a microscope (MP38T, As one, Osaka, Japan) equipped with a microscopic camera (PCM500, As one, Tokyo, Japan). The tissue contour was traced manually on cross-sectional images (total area of section, internal area of epidermis, and non-surface area), and the area was quantified using ImageJ software (v.1.53e; National Institutes of Health, Bethesda, MD, USA).

#### 4.3.3. Total Starch Content

Total starch was measured using the Megazime Total Starch Analysis Kit (K-TSTA). One hundred milligrams of crushed seeds were vortexed with 0.2 mL of 80% ethanol. Then, 3 mL of α-amylase solution was added and mixed well by vortexing. The sample was then incubated at 80 °C for 12 min (vortexing every 4 min). After adding 0.1 mL of amyloglucosidase solution, the sample was vortexed and incubated in a 50 °C bath for 30 min. After mixing 25 mL of distilled water with 25 mL of the supernatant, centrifugation was performed for 10 min at 3000 rpm and 25 °C. Then, 0.1 mL of the supernatant was mixed with 3.0 mL of GOPOD reagent. Incubation was then performed in a 50 °C bath for 20 min. Absorbance (Spectronic 200 UV-Vis spectrophotometer, Thermo Fisher Scientific Inc., Waltham, MA, USA) was measured at 510 nm against a blank solution consisting of water and GOPOD. Total starch was calculated based on the absorbance of a 1 mg/mL D-glucose standard.

#### 4.3.4. α-Amylase Activity

To measure α-amylase activity, 0.3 g rice seeds were sampled at each of phases 1, 2, and 3. The seeds were homogenized and rinsed with 4 mL of ice-cold Na-phosphate buffer (pH 7.0, 0.1 M). After incubation for 24 h at 4 °C, samples were centrifuged at 15,000 g and 4 °C for 20 min, then the supernatant was collected as crude extract. The crude extract was heated for 10 min at 85 °C. Then, 1% soluble starch (Nacalai Tesque Inc., Kyoto, Japan), which was dissolved in acetone buffer at pH 5.6, was added. After 10 min, 2 mL of dinitrosalicylic acid was mixed, and the mixture was boiled for 5 min. The absorbance of the solution was read at 540 nm using a 200 UV-Vis spectrophotometer. The standard curve was obtained by using different concentrations of maltose (Maltose Monohydrate, Nacalai Tesque Inc., Kyoto, Japan) in the range of 0–2.0 µmol L^−1^. α-Amylase activity was measured using the dinitrosalicylic acid method [35].

#### 4.3.5. Total Soluble Sugar Content

Soluble sugars were evaluated in the control and water-deficit treatments in accordance with the methods described by Libron et al. [36]. From each variety of primed and non-primed seeds, 50 mg of seeds were homogenized in 90 °C 80% ethanol. The homogenate was then centrifuged, and the alcohol extract was evaporated until approximately 3 mL remained before being quantitatively transferred to a 25 mL volumetric flask. Aliquots of 0.2 mL were obtained from the sugar extract, which was supplemented with 0.8 mL of distilled water and mixed by vortexing. The solution was then supplemented with 3 mL of Antron reagent and mixed well. The sample was then placed in a water bath at 95 °C for 10 min and cooled in an ice bath. The absorbance of the solution was read at 630 nm using a Spectronic 200 UV-Vis spectrophotometer.

#### 4.3.6. Statistical Analysis

A three-way analysis of variance (ANOVA) was performed using the statistical for software R (version 4.2.2) to determine the effects of variety (“Kasalath” and “Nipponbare”), priming (P or NP), and treatment (well-watered and 15%PEG solution). Tukey’s Honest Significant Difference (HSD) test and Welch’s *t*-test were used to determine the statistical significance of the differences between the mean values (*p* < 0.05).

## 5. Conclusions

This study compared the efficacy of hydropriming in “Kasalath” and “Nipponbare” rice varieties under well-watered and water-deficit conditions. For both cultivars, the time to reach each germination phase was reduced by priming under water-deficit and well-watered conditions. In addition, the size of the coleoptile, plumule, and radicle at each germination phase decreased under water-deficit conditions compared to well-watered conditions, but for primed seeds, it decreased less. α-Amylase and total sugar contents were decreased by osmotic stress, but these decreases were alleviated in primed seeds. Moreover, the increases due to priming were greater in “Kasalath” than in “Nipponbare”, which may be related to the high priming efficiency. Furthermore, the lower content of α-amylase and higher total sugar content in primed “Kasalath” in all phases than in “Nipponbare” could imply that “Kasalath” exhibits highly efficient sugar synthesis. The results obtained in this study may provide a new perspective on priming mechanisms.

## Figures and Tables

**Table 1:** The effects of seed hydropriming on time to 50% germination (T50), mean germination time (MGT), germination uniformity (GU), and germination percentage (GP) under well-watered and water-deficit conditions.

Variety	Water Status	ST	T50 (h)			MGT (h)			GU			GP (%)		
Kasalath	Well-watered	NP	38.38	±	1.61	40.48	±	1.41	11.45	±	6.04	93.00	±	2.74
		P	29.82	±	3.34 **	34.32	±	1.96 **	8.94	±	1.00 ns	99.20	±	1.10 **
	15% of PEG6000	NP	80.15	±	2.13	78.96	±	1.24	14.95	±	1.04	72.60	±	2.51
		P	60.24	±	1.96 **	61.44	±	0.68 **	9.63	±	1.5 **	94.00	±	4.18 **
Nipponbare	Well-watered	NP	69.60	±	3.92	71.40	±	2.55	9.74	±	0.57	92.80	±	2.59
		P	61.25	±	3.56 **	64.08	±	2.10 *	8.29	±	1.05 *	98.40	±	2.04 **
	15% of PEG6000	NP	95.49	±	2.88	95.16	±	2.02	13.29	±	0.90	62.80	±	3.03
		P	76.06	±	4.1 **	78.96	±	0.91 **	11.29	±	1.12 *	88.00	±	5.70 **
*Source*														
Variety (V)			***			***			ns			***		
Priming (P)			***			***			***			***		
Drought (D)			***			***			**			***		
V × P			ns			ns			ns			ns		
V × D			***			***			ns			**		
P × D			***			***			ns			***		
V × P × D			ns			ns			ns			ns		

*, **, and *** indicate a significant difference (*p* < 0.05, *p* < 0.01, and *p* < 0.001, respectively), and data are expressed as mean ± SD (n = 3). ST: seed treatment before germination; NP: not-primed; P: primed; h: hours.

**Table 2:** The effect of hydropriming on changes in the coleoptile area and radicle area of the two rice varieties under well-watered and water-deficit conditions.

Variety	E	ST	Coleoptile (mm^2^)									Radicle (mm^2^)								
			Phase 1			Phase 2			Phase 3			Phase 1			Phase 2			Phase 3		
Kasalath	WW	NP	0.059	±	0.003 ^d^	0.076	±	0.003 ^bc^	0.084	±	0.005 ^bc^	0.026	±	0.002 ^cd^	0.034	±	0.002 ^b^	0.043	±	0.003 ^bc^
		P	0.070	±	0.005 ^bc^	0.086	±	0.004 ^ab^	0.095	±	0.004 ^ab^	0.035	±	0.004 ^a^	0.051	±	0.002 ^a^	0.063	±	0.003 ^a^
	WD	NP	0.045	±	0.005 ^e^	0.054	±	0.005 ^e^	0.063	±	0.005 ^d^	0.017	±	0.001 ^f^	0.021	±	0.002 ^d^	0.028	±	0.005 ^e^
		P	0.055	±	0.003 ^de^	0.067	±	0.004 ^cd^	0.081	±	0.001 ^c^	0.023	±	0.001 ^de^	0.029	±	0.004 ^bc^	0.041	±	0.003 ^bc^
Nipponbare	WW	NP	0.079	±	0.006 ^ab^	0.092	±	0.003 ^a^	0.096	±	0.006 ^ab^	0.029	±	0.001 ^bc^	0.032	±	0.001 ^b^	0.040	±	0.001 ^bc^
		P	0.087	±	0.001 ^a^	0.096	±	0.004 ^a^	0.105	±	0.006 ^a^	0.032	±	0.002 ^ab^	0.037	±	0.002 ^b^	0.048	±	0.002 ^b^
	WD	NP	0.053	±	0.003 ^de^	0.062	±	0.003 ^de^	0.067	±	0.005 ^d^	0.019	±	0.002 ^ef^	0.024	±	0.005 ^cd^	0.032	±	0.003 ^de^
		P	0.061	±	0.003 ^cd^	0.069	±	0.003 ^cd^	0.080	±	0.001 ^c^	0.024	±	0.001 ^cde^	0.030	±	0.002 ^bc^	0.038	±	0.003 ^cd^
*Source*																				
Variety (V)			***			***			***			ns			*			***		
Priming (P)			***			***			***			***			***			***		
Environment (E)			***			***			**			***			***			**		
V × P			ns			ns			ns			ns			**			ns		
V × E			**			*			ns			ns			***			**		
P × E			ns			ns			*			ns			ns			**		
V × P × E			ns			ns			ns			ns			*			ns		

*, **, and *** indicate a significant difference (*p* < 0.05, *p* < 0.01, and *p* < 0.001, respectively). Data are expressed as mean ± SD (n = 3). Tukey’s honestly significant difference (HSD) test at a probability level of 5% was applied. The mean values in each column followed by different lower-case letters are significantly different. E: environment (water status); ST: seed treatment before germination; WW: well-watered; WD: water-deficit treatment (15% PEG6000); NP: not-primed; P: primed.

**Table 3:** Changes in the total starch content and α-amylase activity of rice varieties by hydropriming and water-deficit treatments in phases 1, 2, and 3.

Variety	E	ST	Total Starch Content (%)									α-Amylase Activity (µmol Maltose/mL/min)								
			Phase 1			Phase 2			Phase 3			Phase 1			Phase 2			Phase 3		
Kasalath	WW	NP	48.9	±	1.0 ^ab^	47.9	±	0.4 ^a^	44.7	±	0.2 ^ab^	1.42	±	0.08 ^cd^	1.51	±	0.08 ^de^	1.90	±	0.05 ^d^
		P	48.3	±	1.0 ^abc^	43.9	±	0.9 ^cd^	38.1	±	0.5 ^e^	1.80	±	0.07 ^b^	2.11	±	0.08 ^c^	2.68	±	0.08 ^bc^
	WD	NP	43.3	±	0.4 ^d^	41.8	±	0.3 ^d^	40.1	±	0.2 ^de^	0.84	±	0.06 ^e^	1.02	±	0.04 ^f^	1.37	±	0.15 ^e^
		P	45.9	±	0.9 ^bcd^	43.0	±	0.5 ^d^	38.5	±	0.9 ^e^	1.15	±	0.05 ^de^	1.32	±	0.02 ^ef^	1.91	±	0.04 ^d^
Nipponbare	WW	NP	48.8	±	1.0 ^ab^	46.9	±	0.7 ^ab^	46.7	±	1.3 ^a^	2.58	±	0.08 ^a^	2.70	±	0.06 ^b^	2.93	±	0.00 ^b^
		P	49.8	±	0.5 ^a^	46.1	±	0.2 ^abc^	43.9	±	0.5 ^abc^	2.86	±	0.06 ^a^	3.27	±	0.06 ^a^	3.66	±	0.07 ^a^
	WD	NP	44.9	±	0.1 ^cd^	44.0	±	0.8 ^bcd^	43.0	±	0.4 ^bcd^	1.67	±	0.11 ^bc^	1.78	±	0.11 ^d^	2.05	±	0.05 ^d^
		P	46.0	±	0.6 ^bcd^	43.5	±	0.5 ^cd^	40.9	±	0.7 ^cde^	1.95	±	0.05 ^b^	2.19	±	0.05 ^c^	2.55	±	0.07 ^c^
*Source*																				
Variety (V)			ns			*			***			***			***			***		
Priming (P)			ns			*			***			***			***			***		
Environment (E)			***			***			***			***			***			***		
V × P			ns			ns			ns			ns			ns			ns		
V × E			ns			ns			ns			**			**			**		
P × E			ns			**			**			ns			*			*		
V × P × E			ns			**			*			ns			ns			ns		

*, **, and *** indicate a significant difference (*p* < 0.05, *p* < 0.01, and *p* < 0.001, respectively). Data are expressed as mean ± SD (n = 3). Tukey’s honestly significant difference (HSD) test at a probability level of 5% was applied. Mean values in each column followed by different lower-case letters are significantly different. V: variety; E: environment (water status); ST: seed treatment before germination; K: Kasalath; N: Nipponbare; WW: well-watered; WD: water-deficit treatment (15% PEG6000); NP: not-primed; P: primed.

**Table 4:** Changes in the total soluble sugar content of rice varieties affected by hydropriming and water-deficit treatments in phases 1, 2, and 3.

Variety	E	ST	Total Soluble Sugar Content (mg·g^−1^ DW)								
			Phase 1			Phase 2			Phase 3		
Kasalath	WW	NP	43.40	±	4.01 ^ab^	44.64	±	6.53 ^bc^	66.43	±	5.73 ^bc^
		P	55.72	±	7.17 ^a^	78.92	±	11.94 ^a^	119.50	±	13.05 ^a^
	WD	NP	16.37	±	5.49 ^d^	17.92	±	2.95 ^d^	27.54	±	2.30 ^e^
		P	28.04	±	0.32 ^cd^	39.29	±	2.38 ^c^	62.56	±	0.84 ^bc^
Nipponbare	WW	NP	32.64	±	1.16 ^bc^	35.64	±	4.54 ^c^	48.88	±	7.96 ^cd^
		P	41.67	±	8.11 ^b^	59.53	±	1.52 ^b^	71.57	±	5.86 ^b^
	WD	NP	16.69	±	0.92 ^d^	19.40	±	1.48 ^d^	23.07	±	2.18 ^e^
		P	26.37	±	1.63 ^cd^	31.83	±	1.35 ^cd^	37.81	±	1.45 ^de^
*Source*											
Variety (V)			**			**			***		
Priming (P)			***			***			***		
Environment (E)			***			***			***		
V × P			ns			*			***		
V × E			**			*			**		
P × E			ns			*			*		
V × P × E			ns			ns			ns		

*, **, and *** indicate a significant difference (*p* < 0.05, *p* < 0.01, and *p* < 0.001, respectively). Data are expressed as mean ± SD (n = 3). Tukey’s honestly significant difference (HSD) test at a probability level of 5% was applied. Mean values in each column followed by different lower-case letters are significantly different. V: variety; E: environment (water status); ST: seed treatment before germination; K: Kasalath; N: Nipponbare; WW: well-watered; WD: water-deficit treatment (15% PEG6000); NP: not-primed; P: primed.

## Data Availability

Data will be made available upon request.

## References

[1] Wang T., Sun F. (2023). Integrated drought vulnerability and risk assessment for future scenarios: An indicator based analysis. Sci. Total Environ..

[2] Nyasulu M., Zhong Q., Li X., Liu X., Wang Z., Chen L., He H., Bian J. (2024). Uncovering novel genes for drought stress in rice at germination stage using genome wide association study. Front. Plant Sci..

[3] Seleiman M.F., Al-Suhaibani N., Ali N., Akmal M., Alotaibi M., Refay Y., Dindaroglu T., Abdul-Wajid H.H., Battaglia M.L. (2021). Drought stress impacts on plants and different approaches to alleviate its adverse effects. Plants.

[4] Zhao J., He Y., Li X., Weng X., Feng D., Ying J., Wang Z. (2020). An integrated RNA-Seq and physiological study reveals gene responses involving in the initial imbibition of seed germination in rice. Plant Growth Regul..

[5] Lee S.S., Kim J.H. (2000). Total sugars, alpha-amylase activity, and germination after priming of normal and aged rice seeds. Korean J. Crop Sci..

[6] Li H., Li X., Wang G., Zhang J., Wang G. (2022). Analysis of gene expression in early seed germination of rice: Landscape and genetic regulation. BMC Plant Biol..

[7] Williams J.F., Peterson M.L. (1973). Relations between alpha-amylase activity and growth of rice seedlings 1. Crop Sci..

[8] Damaris R.N., Lin Z., Yang P., He D. (2019). The rice alpha-amylase, conserved regulator of seed maturation and germination. Int. J. Mol. Sci..

[9] Nie L., Song S., Yin Q., Zhao T., Liu H., He A., Wang W. (2022). Enhancement in seed priming-induced starch degradation of rice seed under chilling stress via GA-mediated α-amylase expression. Rice.

[10] Wilson A.M. (1971). Amylase synthesis and stability in crested wheatgrass seeds at low water potentials. Plant Physiol..

[11] Hussain S., Khan F., Hussain H.A., Nie L. (2016). Physiological and biochemical mechanisms of seed priming-induced chilling tolerance in rice cultivars. Front. Plant Sci..

[12] Jumpa T., Phetcharaburanin J., Suksawat M., Pattanagul K., Pattanagu W. (2024). Metabolic profiles and some physiological traits of three rice cultivars differing in salinity tolerance under salinity stress at the germination stage. Asian J. Agric. Biol..

[13] Chen K., Arora R. (2013). Priming memory invokes seed stress-tolerance. Cond. Exp. Bot..

[14] Marthandan V., Geetha R., Kumutha K., Renganathan V.G., Karthikeyan A., Ramalingam J. (2020). Seed priming: A feasible strategy to enhance drought tolerance in crop plants. Int. J. Mol. Sci..

[15] Nakao Y., Asea G., Yoshino M., Kojima N., Hanada H., Miyamoto K., Yabuta S., Kamioka R., Sakagami J.I. (2018). Development of hydropriming techniques for sowing seeds of upland rice in Uganda. Am. J. Plant Sci..

[16] Devika O.S., Singh S., Sarkar D., Barnwal P., Suman J., Rakshit A. (2021). Seed priming: A potential supplement in integrated resource management under fragile intensive ecosystems. Front. Sustain. Food Syst..

[17] Waskow A., Howling A., Furno I. (2021). Mechanisms of plasma-seed treatments as a potential seed processing technology. Front. Phys..

[18] Hernández-Pérez C.A., Gómez-Merino F.C., Spinoso-Castillo J.L., Bello-Bello J.J. (2021). In vitro screening of sugarcane cultivars (*Saccharum* spp. hybrids) for tolerance to polyethylene glycol-induced water stress. Agronomy.

[19] Hellal F.A., El-Shabrawi H.M., Abd El-Hady M., Khatab I.A., El-Sayed S.A.A., Abdelly C. (2018). Influence of PEG induced drought stress on molecular and biochemical constituents and seedling growth of Egyptian barley cultivars. J. Genet. Eng. Biotechnol..

[20] Reyes J.A.O., Casas D.E., Gandia J.L., Parducho M.J.L., Renovalles E.M., Quilloy E.P., Delfin E.F. (2023). Polyethylene glycol-induced drought stress screening of selected Philippine high-yielding sugarcane varieties. J. Agric. Food Res..

[21] Muscolo A., Sidari M., Anastasi U., Santonoceto C., Maggio A. (2014). Effect of PEG-induced drought stress on seed germination of four lentil genotypes. J. Plant Interact..

[22] Shatpathy P., Kar M., Dwibedi S.K., Dash A. (2018). Seed priming with salicylic acid improves germination and seedling growth of rice (*Oryza sativa* L.) under PEG-6000 induced water stress. Int. J. Curr. Microbiol. Appl. Sci..

[23] Kano-Nakata M., Tatsumi J., Inukai Y., Asanuma S., Yamauchi A. (2014). Effect of various intensities of drought stress on δ 13 C variation among plant organs in rice: Comparison of two cultivars. Am. J. Plant Sci..

[24] Tran T.T., Kano-Nakata M., Takeda M., Menge D., Mitsuya S., Inukai Y., Yamauchi A. (2014). Nitrogen application enhanced the expression of developmental plasticity of root systems triggered by mild drought stress in rice. Plant Soil..

[25] Bhattacharjee B., Ali A., Rangappa K., Choudhury B.U., Mishra V.K. (2023). A detailed study on genetic diversity, antioxidant machinery, and expression profile of drought-responsive genes in rice genotypes exposed to artificial osmotic stress. Sci. Rep..

[26] Wolny E., Betekhtin A., Rojek M., Braszewska-Zalewska A., Lusinska J., Hasterok R. (2018). Germination and the early stages of seedling development in *Brachypodium distachyon*. Int. J. Mol. Sci..

[27] Nakao Y., Sone C., Sakagami J.I. (2020). Genetic diversity of hydro priming effects on rice seed emergence and subsequent growth under different moisture conditions. Genes.

[28] Shaik S.S., Carciofi M., Martens H.J., Hebelstrup K.H., Blennow A. (2014). Starch bioengineering affects cereal grain germination and seedling establishment. J. Exp. Bot..

[29] Farooq M., Wahid A., Ahmad N., Asad S.A. (2010). Comparative efficacy of surface drying and re-drying seed priming in rice: Changes in emergence, seedling growth and associated metabolic events. Paddy Water Cond..

[30] Kambona C.M., Koua P.A., Léon J., Ballvora A. (2023). Stress memory and its regulation in plants experiencing recurrent drought conditions. Theor. Appl. Genet..

[31] Lemmens E., Deleu L.J., De Brier N., De Man W.L., De Proft M., Prinsen E., Delcour J.A. (2019). The impact of hydro-priming and osmo-priming on seedling characteristics, plant hormone concentrations, activity of selected hydrolytic enzymes, and cell wall and phytate hydrolysis in sprouted wheat (*Triticum aestivum* L.). ACS Omega.

[32] Adachi Y., Sugiyama M., Sakagami J.I., Fukuda A., Ohe M., Watanabe H. (2015). Seed germination and coleoptile growth of new rice lines adapted to hypoxic conditions. Plant Prod. Sci..

[33] Ren M., Tan B., Xu J., Yang Z., Zheng H., Tang Q., Wang W. (2023). Priming methods affected deterioration speed of primed rice seeds by regulating reactive oxygen species accumulation, seed respiration and starch degradation. Front. Plant Sci..

[34] Purbajanti E.D., Kusmiyati F., Fuskhah E., Rosyida R., Adinurani P.G., Vincēviča-Gaile Z. (2019). Selection for drought-resistant rice (*Oryza sativa* L.) using polyethylene glycol. IOP Conference Series: Earth and Conditional Science.

[35] Bernfeld B. (1955). Amylases α and α. Methods Enzymol..

[36] Libron J.A., Putri H.H., Bore E.K., Chepkoech R., Akagi I., Odama E., Goto K., Tamaru S., Yabuta S., Sakagami J.I. (2024). Halopriming in the submergence-tolerant rice variety improved the resilience to salinity and combined salinity-submergence at the seedling stage. Plant Physiol. Biochem..

